# Association of the 5HTR2A gene with suicidal behavior: CASE-control study and updated meta-analysis

**DOI:** 10.1186/1471-244X-13-25

**Published:** 2013-01-12

**Authors:** Thelma Beatriz González-Castro, Carlos Tovilla-Zárate, Isela Juárez-Rojop, Sherezada Pool García, Martha Patricia Velázquez-Sánchez, Alma Genis, Humberto Nicolini, Lilia López Narváez

**Affiliations:** 1 División Académica de Ciencias de la Salud, Universidad Juárez Autónoma de Tabasco, Villahermosa, Tabasco, México; 2 División Multidisciplinaria de Comalcalco, Universidad Juárez Autónoma de Tabasco, Comalcalco, Tabasco, México; 3 Hospital General de Comalcalco, Tabasco, Secretaría de Salud, Comalcalco, Tabasco, México; 4 Grupo de Estudios Médicos y Familiares Carracci, México, DF, México; 5 CIGEN, Centro de Investigación Genómica, Comalcalco, Tabasco, México; 6 Hospital General de Yajalón, Yajalón, Chiapas, México; 7 División Académica Multidisciplinaria de Comalcalco, Ranchería Sur, Cuarta Sección, C.P. 86650, Comalcalco, Tabasco, México

## Abstract

**Background:**

The polymorphism rs6313 (T102C) has been associated with suicidal behavior in case–control and meta-analysis studies, but results and conclusions remain controversial. The aim of the present study was to examine the association between T102C with suicidal behavior in a case–control study and, to assess the combined evidence – this case–control study and available data from other related studies – we carried out a meta-analysis.

**Methods:**

We conducted a case–control study that included 161 patients with suicide attempts and 244 controls; we then performed a meta-analysis. The following models were evaluated in the meta-analysis: A) C allele vs T allele; B) T allele vs C allele; C) Caucasian population, D) Asian population, and E) suicide attempters with schizophrenia.

**Results:**

We found an association between attempted suicide and control participants for genotype (χ2=6.28, p=0.04, df=2) and allele (χ2=6.17, p=0.01, df=1, OR 1.48 95% IC: 1.08-2.03) frequencies in the case–control study. The meta-analysis, comprising 23 association studies (including the present one), showed that the rs6313 polymorphism is not associated with suicidal behavior for the following comparisons:T allele vs C allele (OR: 1.03; 95% CI 0.93-1.13; p(Z)=0.44); C allele vs T allele: (OR:0.99; 95% CI: 0.90-1.08; p(Z)=0.22); Caucasians (OR:1.09; 95% CI: 0.96-1.23), and Asians (OR:0.96; 95% CI: 0.84-1.09).

**Conclusion:**

Our results showed association between the rs6313 (T102C) polymorphism and suicidal behavior in the case–control study. However, the meta-analysis showed no evidence of association. Therefore, more studies are necessary to determine conclusively an association between T102C and suicidal behavior.

## Background

Suicidal behavior is a major health problem worldwide. Several recent studies have been carried out that support a possible relationship between genetic factors and suicidal behavior [1,2]. Historically, evidence for the involvement of serotonin (5-HT) in suicide originated from findings of low 5-hydroxyindoleacetic acid concentration (5-HIIA) in cerebrospinal fluid (CSF) of depressed suicide attempters and in brain stems of completed suicides [3-5]. These studies provided evidence for altered serotonergic neural transmission in the pathogenesis of suicidal behavior [6,7]. In consequence, genes pertaining to the serotonergic system have been proposed as candidates to establish biological correlates between suicidal behavior and the serotonergic system [8-12].

Fourteen known serotonin receptor subtypes are involved in serotonin action [13]. One candidate gene in the study of suicidal behavior is the gene encoding the serotonin 2A receptor. There is evidence that the density of serotonin 2A receptors is upregulated in parietal cortical regions of depressed suicide victims and this increase has been suggested to be at least partly regulated genetically [14]. The 5HTR2A gene is located on chromosome 13 (13q14-q21); it spans 20 kb and contains 3 exons; more than 200 SNPs have been identified along the gene [13,15]. Mainly, the polymorphisms A-1438G (rs6311), T102C (rs6313) and His452Tyr (rs6314) have been studied in relation to suicidal behavior. Moreover, a recent report has studied all SNPs in 5HTR2A [16]. One of the most remarkable single nucleotide polymorphisms of the 5HTR2A gene is T102C (rs6313). Several genetic studies have shown an association between 5HTR2A and suicidal behavior [17,18]. Similarly, this common variant has been studied not only in relation to suicidal behavior, but also in association with other diseases such as schizophrenia [19-21], alcoholism [22], and depression [23].

To our knowledge, up to the present moment, there are no association studies of 5HTR2A and suicidal behavior in the Mexican population. Therefore, we examined the association between 5HTR2A and suicidal behavior in this population. This work focuses on investigating the genetic basis of suicide attempters by assessing the rs6313 genotype. Finally, we used the combined evidence to carry out a meta-analysis of all published data.

## Methods

### Case–control study

A total of 161 patients were consecutively recruited from the outpatient service of the General Hospital of Comalcalco in the state of Tabasco, Mexico. These patients had attempted suicide between January and December 2011. In addition, 247 unrelated controls participated in this study. They were recruited from the Blood Donor Center of the General Hospital of Comalcalco and from the general population of the Comalcalco city area in the state of Tabasco, México. Subjects were physically healthy on medical evaluation; none manifested psychiatric problems, as assessed in brief interviews by psychiatrists. To reduce ethnic variation and stratification effects, only Mexican subjects descending from Mexican parents and grandparents participated in this study. However, no structured methods were used to assess genetic heterogeneity.

### Ethics statement

After they were given a verbal and written explanation of the research objectives, all subjects signed an informed consent to participate in the study; they did not receive any economical remuneration. This study complied with the principles convened in the Helsinki Declaration. In addition, this study was approved by the DAMC-UJAT Ethics and Research Committee (P.O.A. 20110237).

### Clinical evaluation

Suicide attempt patients were evaluated by a trained psychiatrist or clinical psychologists with at least a master's degree level, using the Structured Clinical Interview for DSM-IV Axis-I and II diagnoses, in the Spanish version. We defined suicide attempt as a self-harm behavior with at least some intent to end one’s life [24]. Subjects were excluded when self-injury behaviors were determined to have no suicidal intention or ideation, based on the Assessment of suicidal intention: the Scale for suicide ideation in the Spanish version [25].

### Genotype assays

Genomic DNA was extracted from peripheral blood leukocytes using a modified version of the protocol by Lahiri [26]. The 5HTR2A T102C (rs6313) genotypes were analyzed in all patients using a polymerase chain reaction (PCR) end-point method. The final volume of the PCR reaction was 5 μL and consisted of 20 ng genomic DNA, 2.5 Fluorescence Labeling (FL) TaqMan Master Mix, and 2.5 FL 20x Assay. The amplification was performed in 96-well plates using the TaqMan Universal Thermal Cycling Protocol. After the PCR end-point was reached, fluorescence intensity was measured with the 7500 Real-Time PCR system using SDS v2.1 software (Applied Biosystems). All genotyping was performed blind to patient outcome. As a quality control in our genotyping analyses we used random blind duplicates.

### Statistical analysis

Hardy-Weinberg equilibrium was tested using Pearson's goodness-of-fit chi-squared test. Chi-squared test or Fisher's Exact test was used to compare genotype and allele frequencies between groups. The power to detect associations given the sample size was analyzed by using the Quanto 1.2 software. The power of the analysis was 0.43 (minor allele frequency= 0.3, type of model: dominant, effect size: 1.5). The level of significance was set at p=0.05.

### Meta-analysis

This meta-analysis followed the Preferred Reporting Items for Systematic Reviews and Meta-Analyses (PRISMA) criteria [27].

### Identification and selection of publications

A literature search was performed that covered from 1997 to 2012. Relevant publications were identified using the following search terms in Medline, Ebsco and Web of Science databases: “HTR2A and suicidal behavior”, “HTR2A and suicide”, “rs6313 and suicide”, “5-hydroxytryptamine-receptor 2A” and “serotonin receptor 2A”. These words were combined to retrieve the summaries. The search also implicated the review of the bibliography cited at the end of various research articles to identify additional papers not covered by the electronic search of abstracts. The publications had to fulfill the following selection criteria: (1) to be studies on the relationship between rs6313 and suicidal behavior (attempted, ideation and completed suicide), (2) to be published in peer-reviewed journals, (3) to contain independent data, (4) to be case–control association studies in which the frequencies of three genotypes were clearly stated or could be calculated, (5) the use of healthy individuals as controls, and (6) to provide sufficient data to enable the estimation of an odds ratio with 95% confidence interval.

Two investigators (González-Castro TB and Tovilla-Zárate CA) working independently screened each of the titles, abstracts and full texts to determine their inclusion. The results were compared and disagreements were resolved by consensus.

### Data extraction

The same authors mentioned in the last paragraph carefully extracted the information from all the included publications; they worked in an independent manner and in accordance with the inclusion criteria listed above. The following data were obtained from each of the studies: authors, year of publication, region, number of cases and controls, age, and ethnical origin of the participants. The outcomes of the meta-analysis were built by taking into consideration the following categories: a) exposed sick, b) exposed not-sick, c) not-exposed sick and d) not-exposed not-sick. The “sick” term refers to subjects exhibiting suicidal behavior and the “exposed” term to the allele of risk.

### Data analysis

For the meta-analysis procedures, we used the EPIDAT 3.1 program (http://dxsp.sergas.es). This software is freely available for epidemiologic analysis of tabulated data. Data were analyzed with the random-effects model following the reports in the literature [2,28,29]. Sample heterogeneity was analyzed with the Dersimonian and Laird’s Q test. Q test results were complemented with graphs to help the visualization of those studies favoring heterogeneity. The results of the meta-analysis are expressed as an odds ratio (OR). To address the problem of publication bias, funnel plots were calculated with the EPIDAT 3.1 software. This plotting standardizes the effect of each of the published studies on the vertical axis and its correspondent precision on the horizontal axis. Finally, a chi-squared (χ^2^) analysis was used to calculate the Hardy-Weinberg equilibrium to assess genotype distribution.

## Results

### Case–control study

In the present study a total of 161 patients (61 males, 100 females) were recruited; mean age was 25.5 (9.56) years old (range: 14–56 years of age). Control subjects consisted of 244 volunteers (82 males, 162 females); mean age was 33.1 (13.0) years old (range: 14–61 years of age). Both groups (cases p=0.06 and controls p=0.06) showed Hardy-Weinberg equilibrium for the analyzed genetic variability. From the 161 suicide attempters, 11 (7%) exhibited the T102T genotype, 80 (50%) the T102C, and 70 (43%) the C102C type. On the other hand, in the control group, 9 individuals (4%) presented the T102T genotype, 100 (41%) the T102C type, and 135 (55%) the C102C type. We observed significant differences in genotype (χ2=6.28, p=0.04, df=2) and allele (χ2=6.17, p=0.01, df=1, OR 1.48 95% IC: 1.08-2.03) frequencies between patients and the control group.

### Meta-analysis study

**Figure 1:**
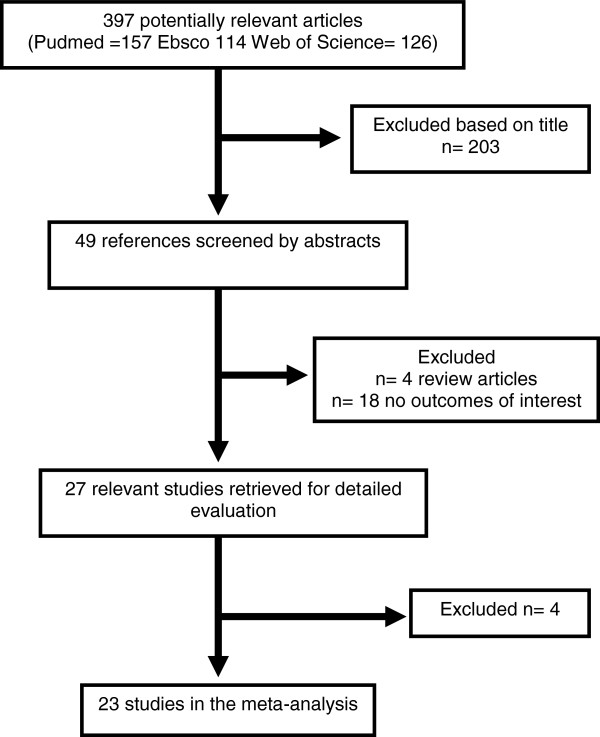
Flow-chart showing the different phases of the meta-analysis.

The selection process in this study is detailed in Figure 1. With regard to the literature search, a total of 397 studies were identified, but only 23 were used in this meta-analysis, including our case–control study [14,20-23,30-40]; this involved 2566 cases and 3989 controls (Table 1). In addition, 612 cases from a more recent meta-analysis and 1129 controls were incorporated as well [41]. Overall, the meta-analysis included 13 studies in Caucasian populations, 6 studies comprising Asian populations and 4 from other ethnic origins.

**Table 1:** Descriptive characteristics from 23 studies on the role of theT102C polymorphism of the 5HTR2A gene in suicidal behavior

**Study (Year)**	**Sample Size n (Cases-Controls)**	**Location**	**Diagnosis**	**Number of C alleles**	**Gender (males/females)**		**Mean age (years)**	
					**Cases**	**Controls**	**Cases**	**Controls**
Tan (2002)	47/94	Singapore	SA with schizophrenia	32	47	94		
Pooley (2003)	129/329	United Kingdom Caucasians	SA	154	52/77	138/191	38	38
Chen (2001)	471/523	Hong Kong	SA with schizophrenia	193	314	308/215		
Saiz (2008)	193/420	Asturias in Northern Spain	SA	196	70/123	216/204	35	40
Du (2000)	42/131	Ottowa	SA with major depression	99	48/30	61/70	40	36
Wrsozek (2011)	38/118	Polish Caucasians	SA with alcohol-dependent	33	26/12	81/29	>18	>18
Khait (2005)	52/63	European Caucasians	SA with depression	54	30/24	12/51	37	40
Correa (2007)	42/85	Brazilian	SA with schizophrenia	83	23/19	45/40		
Vaquero-Lorenzo (2008)	441/410	Spanish Caucasians	SA	472	161/280	237-173	38	36
Du (1999)	24/31	Budapest, Hungary	SC with depression	32	24	31	45	50
Bondy (2000)	131/125	Caucasians Germany	SA with major depression	142	91/40	60/75	46	47
Zhang (2008)	297/303	Shangai	SA with schizophrenia, anxiety and organic mental disorder	229	171/126	217/112	46	43
Preuss (2000)	45/117	Caucasians Germany	SA with alcohol-dependence	52	45	56/61		47
Preuss (2000)	62/117	Caucasians Germany	SI with alcohol-dependence	66	62	56/61		47
Ertugrul (2004)	71/26	Caucacians and African-americans	SA with schizophrenia	49	26/12	81/29		
Crawford (2000)	78/131	Caucasians Australian	SC	73	68	95		
Crawford (2000)	68/95	Caucasians Australian	SI	99	78	131		
Turecki (1999)	56/126	Montreal	SC	68	56	126	36	33
Ono (2001)	151/163	Japanese	SC	157	106/45	108/55	47	47
Zhang (1997)	15/150	Japanese	SA with mood disorders	21	15	65/85	33	37
Tsai (1999)	61/96	Taipei	SA with mood disorders	49	61	96		71
Arias (2001)	33/164	Spanish	SA	42	33	83/71	55	41
Tovilla (2012)	161/244	Mexican	SA	220	61/100	82/162	25	33

SA: Suicide attempters; SC: Suicide completers; SI: Suicide ideation.

We measured the Hardy-Weinberg equilibrium in all genotyped populations. Both patients and controls were in equilibrium (p>0.05), with the exception of the control group in Du et al. (p=0.004), Turecki et al. (0.009) and Crawford et al. (0.01) [14,30,35], as well as the patients in Zhang et al. (p=0.04) [38]. When we explored all populations in a combined way we still encountered them in equilibrium with p=0.19 and p=0.92 for controls and cases, respectively.

**Figure 2:**
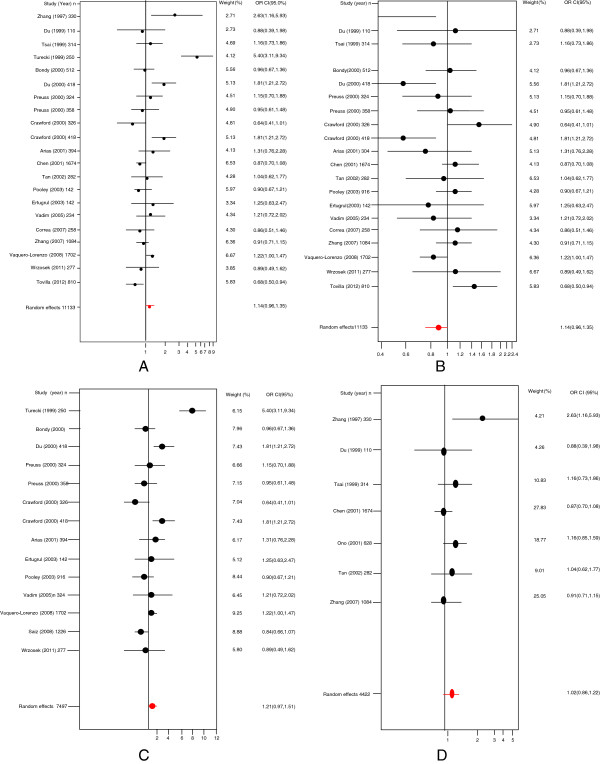
**Odds ratios and forest plots of all models in overall studies. A**) C allele vs T allele; **B**) T allele vs C allele; **C**) Caucasian population, and **D**) Asian population.

**Figure 3:**
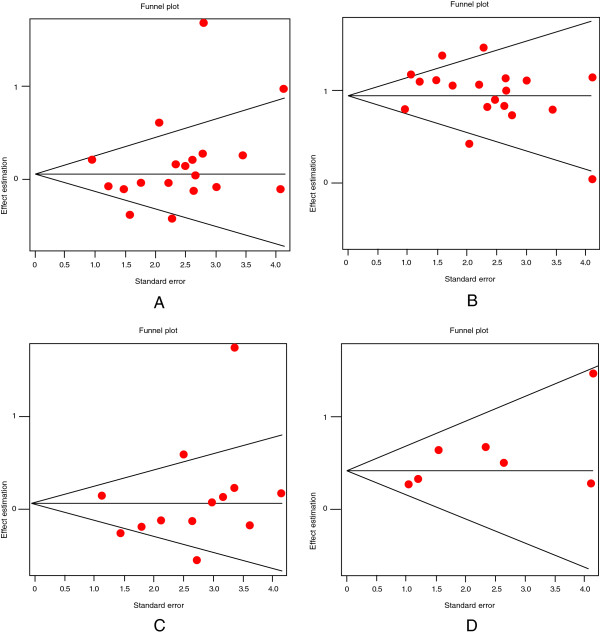
**Egger's funnel plot indicating publication bias for studies with suicidal behavior. A**) C allele vs T allele; **B**) T allele vs C allele; **C**) Caucasian population, and **D**) Asian population.

Figure 2 shows the pooled OR derived from all studies indicating a non-significant association of the C allele in the T102C polymorphism with suicidal behavior (Random effects: OR:1.14; 95% CI 0.96-1.35; p(Z)=0.07). We observed heterogeneity in all studies (Q=75.03 df=20; p=<0.0001). The Egger's test provided no evidence of publication bias (t=1.37, df=19; p=0.18) (Figure 3A). Therefore, we carried out a second analysis, which excluded studies based on the heterogeneity curve and sensitivity analysis. As a result, the reports by (Crawford et al. [35], Zhang et al. [38], Du et al. [30], Turecki et al. [14] and ours were excluded. However, we could not find an association either (OR: 1.03; 95% CI 0.93-1.13; p(Z)=0.44).

Given that our results in the association study suggested an association with the T allele, we performed a meta-analysis using the allelic model for this allele (Table 2). We did not encounter any association between the T allele and suicidal behavior (Random effects; OR: 0.87; 95% CI 0.73-1.03; p(Z)=0.07). We also found heterogeneity in all studies (Q=75.03 df=20; p=<0.0001). The Egger's test indicated no evidence of publication bias (t=−1.37, df=19; p=0.18; Figure 3B). A second analysis was performed, which only included studies based on the heterogeneity curve and sensitivity analysis (Random effects: OR: 0.99; 95% CI: 0.90-1.08; p(Z)=0.22). The Egger's test gave no evidence of publication bias (t=−0.1726, df=12; p=0.86).

**Table 2:** Meta-analysis of case–control studies on the role of the T and C alleles in the T102C polymorphism of the 5HTR2A gene in individuals with suicidal behavior

**References**	**Number of T alleles**		**Number of C alleles**		**Odds Ratio (95%IV)**	
	**Cases**	**Controls**	**Cases**	**Controls**	**T Allele**	**C Allele**
Correa	91	41	83	43	1.14 (0.68 - 1.93)	1.14 (0.68 - 1.93)
Wrzosek	24	87	33	133	1.11(0.61 - 2.00)	1.11( 0.61 – 2.00)
Zhang	287	303	229	265	1.09 (0.86 - 1.39)	1.09 ( 0.86 - 1.39)
Vadim	54	69	54	57	0.82 (0.49 – 1.38)	0.82 ( 0.49 - 1.38)
Du	57	134	99	128	0.55 (0.36 - 0,8254)	1.13 (0.50 - 2.53)
Vaquero-Lorenzo	410	422	472	398	0.81(0.67- 0.99)	0.81 ( 0.67- 0.99)
Du	16	19	32	43	1.13( 0.50 - 2.53)	0.55 ( 0.36 – 0.82)
Bondy	120	112	142	138	1.04 ( 0.73- 1.47)	1.04 (0.73 - 1.47)
Zhang	9	159	21	141	1.09 (0.86 - 1.39)	1.09 (0.86 - 1.39)
Arias	24	141	42	187	0.75 ( 0.43 - 1.30)	0.38 ( 0.16 - 0.85)
Tsai	73	122	49	70	0.85 ( 0.53 - 1.36)	0.85( 0.53 - 1.36)
Turecki	40	108	68	34	0.18 (0.10 - 0.32)	0.18 ( 0.10 - 0.32)
Preuss	38	107	52	127	0.86 ( 0.53 - 1.41)	0.55 ( 0.36 - 0.82)
Preuss	58	107	66	127	1.04 ( 0.67 - 1.61)	1.04 (0.67 – 1.61)
Crawford	63	68	73	122	1.54 ( 0.98 - 2.42)	1.54 ( 0.98 - 2.42)
Crawford	57	134	99	128	0.55 ( 0.36 - 0.82)	0.55 ( 0.36 – 0.82)
Ertugrul	39	27	49	27	0.79 ( 0.40 - 1,56)	0.79 (0.40 - 1.56)
Tan	62	126	32	62	0.95 ( 0.56 - 1.60)	0.95 (0.56 - 1.60)
Pooley	104	249	154	409	1.10 ( 0.82 - 1.48)	1.10 (0.82 - 1.48)
Chen	435	694	193	352	1.14 (0.92- 1.41)	1.14 (0.92 - 1.41)
Tovilla	102	118	220	370	1.45 ( 1.06 - 1.98)	1.45 (1.06 - 1.98)
Ono	145	169	157	157		
Saiz	190	378	196	462		
Random effects	2498	3894	2615	3980	0.87 ( 0.73 – 1.03)	0.87(0.73 - 1.03)

In addition, we performed an analysis in two population groups: Caucasian (Fixed effects; OR: 1.14; 95% CI: 1.03-1.25, Random effects OR: 1.21; 95% CI: 0.97-1.57 with heterogeneity; Egger's test Figure 3C, OR: 1.09; 95% CI: 0.96-1.23 without heterogeneity; Table 3) and Asian (OR: 0.96; 95% CI: 0.84-1.09 without heterogeneity; Egger's test Figure 3D). Again in this case, we could not find any association with suicidal behavior. Finally, in an analysis including only studies of suicide attempt patients with schizophrenia, the results were also negative (Random effects; OR: 0.91; 95% CI: 0.79-1.06 without heterogeneity).

**Table 3:** Results of Caucasian and Asian populations with suicidal behavior

	**OR**	**CI (95%)**
Overall studies with heterogeneity	1.14	(0.96,1.35)
Overall studies without heterogeneity	1.03	(0.93,1.13)
Caucasians with heterogeneity	28.3704	(16.91,47.57)
Caucasians without heterogeneity	1.09	(0.96,1.23)
Asians with heterogeneity	1.02	(0.86,1.22)
Asians without heterogeneity	0.96	(0.84,1.09)

## Discussion

In this study, we explored the association of the T102C (rs6313) polymorphism of the 5-HTR2A gene with suicidal behavior. First, a case–control study was conducted. Subsequently, we performed a meta-analysis to assess the current evidence of association between rs6313 and suicidal behavior. In the first part, we found an association between rs6313 and suicidal behavioral in a Mexican sample. To our knowledge, this is the first study addressing the genetic association between the T102C allele and suicidal behavior in a Mexican population.

The genetic analysis revealed a slight preponderance of the T allele in the cases group. In 2000, Du et al. described an association between the 102C allele and ideation suicide [30]. Since 2000, growing interest on this issue prompted a variety of studies in Caucasian and Asian populations dealing with 5HTR2A and suicidal behavior. Although many studies have reported this association [21,22,33,42], there are other reports that have not encountered an association of either the T or C allele in rs6313 of 5HTR2A [20,23,31,32,35,38,43] with suicidal behavior. The slight preponderance of the T allele is in agreement with other reports in the literature. In 1999, Tsai et al. [34] reported that the allele frequency of 102T is higher than that of 102C in both patient and control groups. Recently, Saiz et al. [36] found an excess of the 102T allele in a group of 193 patients and a control group of 420 individuals when comparing non-impulsive suicide attempts with impulsive suicide attempts. This evidence suggests that the rs6313 polymorphism of the 5-HTR2A gene may be involved in suicidal behavior. However, no conclusive outcomes have yet been attained. In agreement, our results in the Mexican population suggest that allele 102T of 5HTR2A could be associated with suicidal behavior.

There are several possible explanations for the discrepancies in the results regarding this association. First, there are differences in the populations of patients. For example, Du et al. [30] and other authors [13,23] have studied patients with major depression and suicidal behavior; Wrzosek et al. studied patients with attempted suicides and alcohol dependence [22]; other studies have analyzed suicidal behavior in patients with schizophrenia [20], and finally, there are studies such as ours that have analyzed only suicide attempters [8,31,36]. Second, there are differences in genetic heterogeneity. Several authors have shown that allele frequencies of the T102C polymorphism may be ethnic-dependent [34,36,44]. However, our sample was made of a relatively homogenous Mexican population, because only patients of the state of Tabasco with parents and grandfathers born in Tabasco were included in this study. In consequence, we considered necessary to carry out a meta-analysis of all the published evidence concerning rs6313 and suicidal behavior.

In the current meta-analysis we utilized the allelic model and ethnicity groups. There are two previous meta-analyses -the last including 25 studies- that have explored the association between rs6313 and suicidal behavior [41,45]. Both meta-analyses reported no association between rs6313 and suicidal behavior. However, several additional case–control studies concerning this relationship and using larger samples have been recently reported, but they are not included in the above-mentioned meta-analyses. Therefore, to get a more comprehensive estimation we included our case–control study.

We performed a meta-analysis for the C (OR: 1.14; 95% CI 0.96-1.35) and T (OR: 0.99; 95% CI: 0.90-1.08) alleles separately; however, no association was observed. The results suggest no association between rs6313 (T102C) and suicidal behavior. One possibility for this lack of association may lie in the different criteria used in the selection of patients. In addition, samples sizes of several of the studies included in our meta-analysis are in the low range compared with genetic studies for other diseases [7,46].

There is the possibility that the C allele may be regarded as a factor risk in the Caucasian population, because in a first analysis we observed an association when we considered a fixed effects with heterogeneity. However, we adopted the random effects, which accounts for additional sources of inter-study variation when heterogeneity exists. This model is more conservative than the fixed effects, since the latter assumes the same true genetic effects, whereas the former assumes normally distributed effects and parametrizes inter-studies variation [41]. When we performed a second analysis, in which the studies giving rise to heterogeneity were discarded, no association was observed between rs6313 and suicidal behavior in a Caucasian population. Therefore, future studies on suicidal behavior comprising larger samples are important to determine this association. Similarly, the accuracy of the phenotype definition in the association studies is a relevant issue [41]. However, it is necessary to take into consideration other aspects such as different inheritance patterns and gene X environment interaction [16].

Finally, we recognize some limitations in the present study; 1) the sample size of the case–control study is small and may not have sufficient power to detect an association between suicidal behavior and small effects polymorphism. In relation to our meta-analysis, there are also some considerations to bear in mind: first; included studies were limited to published articles; second, we did not perform sub-analyses including attempted or completed suicide; third, we did not carry out a subgroup analysis based on gender, and fourth, we did not report psychopathologies related to suicide attempters.

## Conclusions

In conclusion, we encountered a significant association between the T102C polymorphism and suicidal behavior in a Mexican population. However, this result was not observed in the meta-analysis. Nonetheless, our meta-analysis provides pooled data on a substantial number of cases and controls that may provide a better understanding in the association between rs6313 and suicidal behavior. However, more comprehensive studies and larger samples are necessary to determine conclusively the presence of this association.

## Abbreviations

PRISMA: Preferred Reporting Items for Systematic Reviews and Meta-Analyses; DSM-IV: Diagnostic and Statistical Manual of Mental Disorders-IV; SA: Suicide attempters; SC: Suicide completers; SI: Suicide ideation.

## Competing interests

The authors declare not to have any competing interests.

## Authors’ contributions

TZC and GCTB conceived the study, participated in its design, and helped to draft the manuscript. TZC, JRI, LNL and NH helped to perform the statistical analysis and to draft the manuscript. PGS and VSMP recruited participants and helped with data integration and analysis. GA, TZC and HN coordinated and supervised the integration of data. All authors read and approved the final manuscript.

## Pre-publication history

The pre-publication history for this paper can be accessed here:

http://www.biomedcentral.com/1471-244X/13/25/prepub
